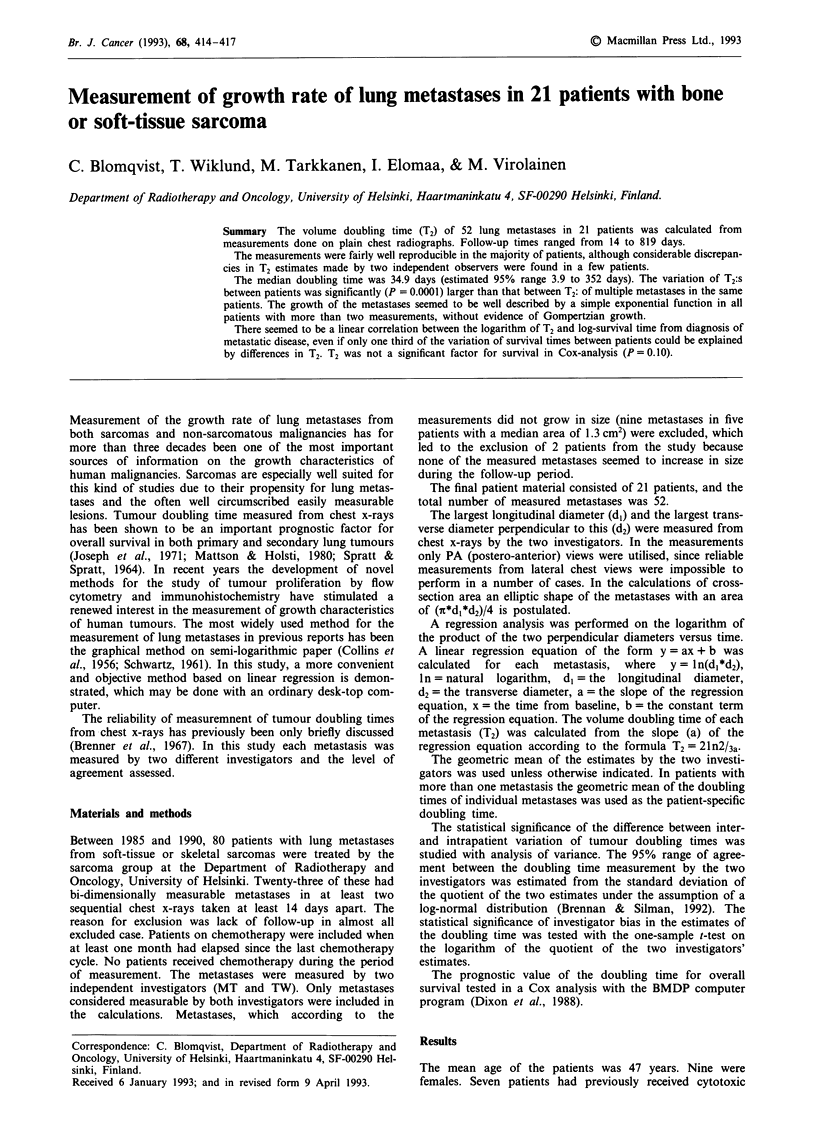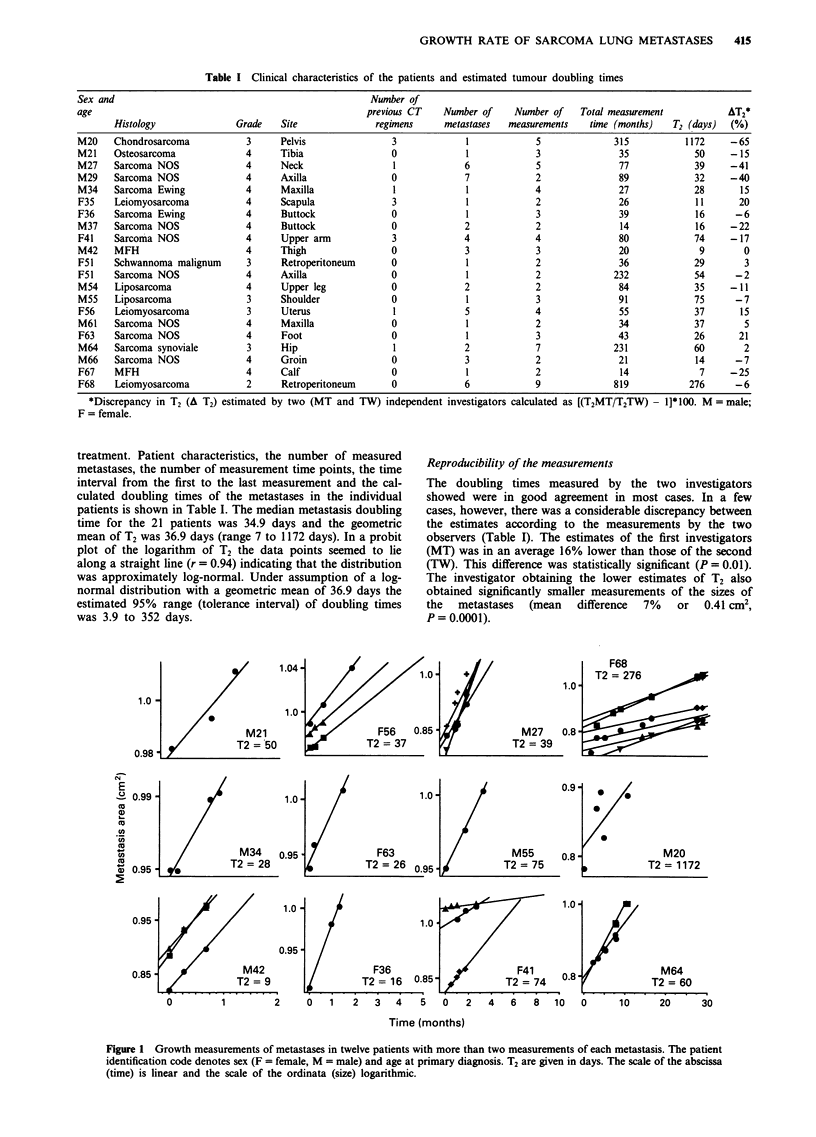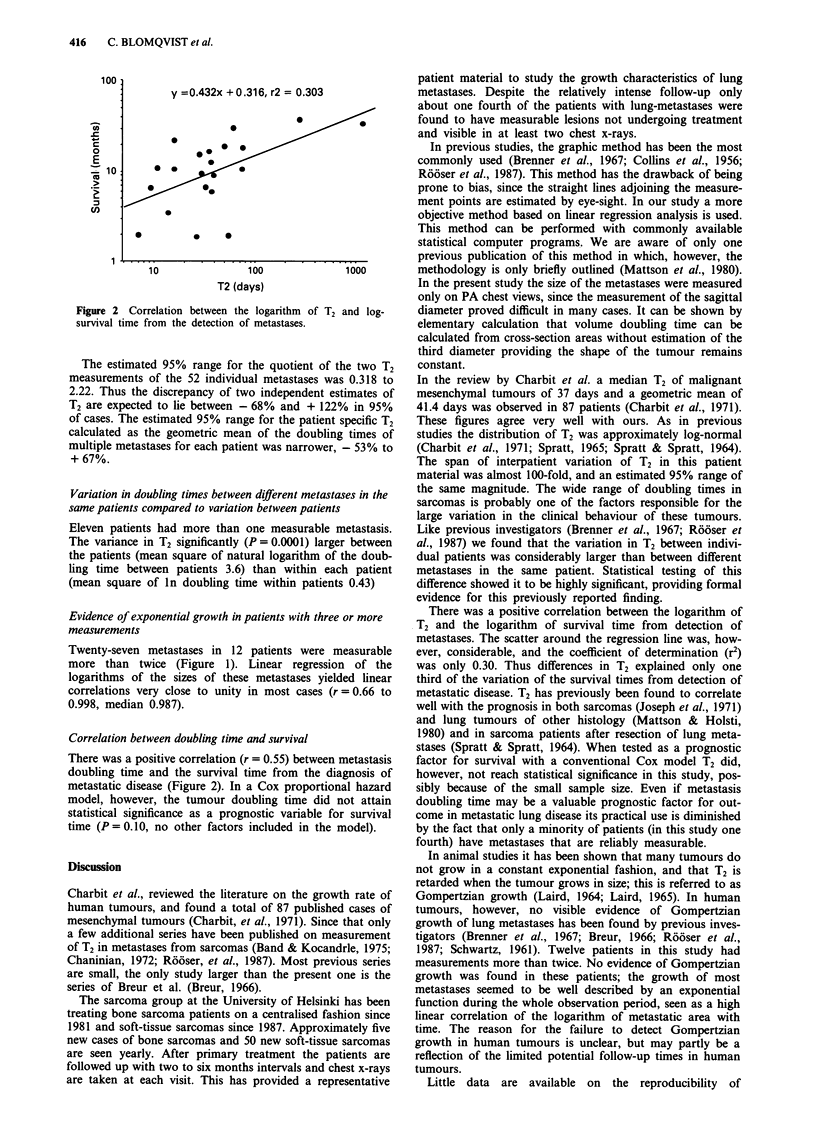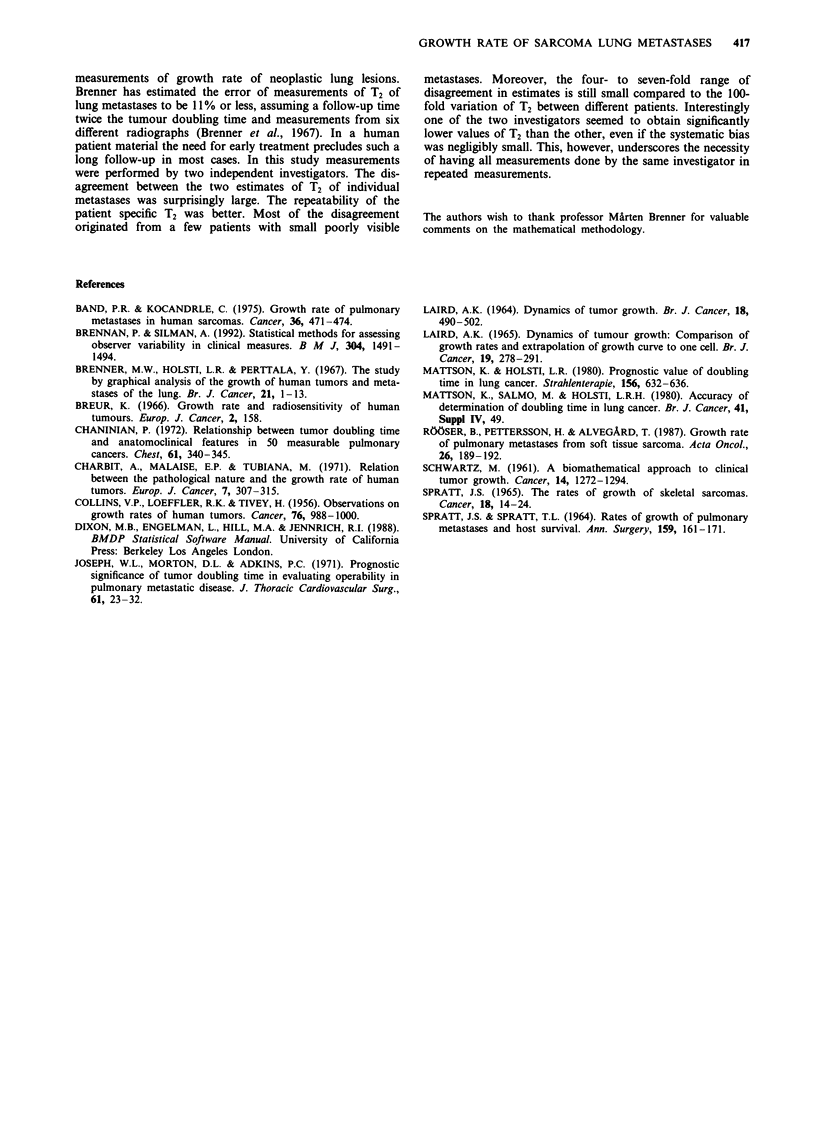# Measurement of growth rate of lung metastases in 21 patients with bone or soft-tissue sarcoma.

**DOI:** 10.1038/bjc.1993.351

**Published:** 1993-08

**Authors:** C. Blomqvist, T. Wiklund, M. Tarkkanen, I. Elomaa, M. Virolainen

**Affiliations:** Department of Radiotherapy and Oncology, University of Helsinki, Finland.

## Abstract

The volume doubling time (T2) of 52 lung metastases in 21 patients was calculated from measurements done on plain chest radiographs. Follow-up times ranged from 14 to 819 days. The measurements were fairly well reproducible in the majority of patients, although considerable discrepancies in T2 estimates made by two independent observers were found in a few patients. The median doubling time was 34.9 days (estimated 95% range 3.9 to 352 days). The variation of T2:s between patients was significantly (P = 0.0001) larger than that between T2: of multiple metastases in the same patients. The growth of the metastases seemed to be well described by a simple exponential function in all patients with more than two measurements, without evidence of Gompertzian growth. There seemed to be a linear correlation between the logarithm of T2 and log-survival time from diagnosis of metastatic disease, even if only one third of the variation of survival times between patients could be explained by differences in T2. T2 was not a significant factor for survival in Cox-analysis (P = 0.10).


Br. J. Cancer (1993), 68, 414-417 © Macmillan Press Ltd., 1993

### Measurement of growth rate of lung metastases in 21 patients with bone or soft-tissue sarcoma

C. Blomqvist, T. Wiklund, M. Tarkkanen, I. Elomaa, & M. Virolainen

*Department of Radiotherapy and Oncology, University of Helsinki, Haartmaninkatu 4, SF-00290 Helsinki, Finland.*

**Summary** The volume doubling time ($T_2$) of 52 lung metastases in 21 patients was calculated from measurements done on plain chest radiographs. Follow-up times ranged from 14 to 819 days.

The measurements were fairly well reproducible in the majority of patients, although considerable discrepancies in $T_2$ estimates made by two independent observers were found in a few patients.

The median doubling time was 34.9 days (estimated 95% range 3.9 to 352 days). The variation of $T_2$:s between patients was significantly ($P = 0.0001$) larger than that between $T_2$: of multiple metastases in the same patients. The growth of the metastases seemed to be well described by a simple exponential function in all patients with more than two measurements, without evidence of Gompertzian growth.

There seemed to be a linear correlation between the logarithm of $T_2$ and log-survival time from diagnosis of metastatic disease, even if only one third of the variation of survival times between patients could be explained by differences in $T_2$. $T_2$ was not a significant factor for survival in Cox-analysis ($P = 0.10$).

Measurement of the growth rate of lung metastases from both sarcomas and non-sarcomatous malignancies has for more than three decades been one of the most important sources of information on the growth characteristics of human malignancies. Sarcomas are especially well suited for this kind of studies due to their propensity for lung metastases and the often well circumscribed easily measurable lesions. Tumour doubling time measured from chest x-rays has been shown to be an important prognostic factor for overall survival in both primary and secondary lung tumours (Joseph *et al.*, 1971; Mattson & Holsti, 1980; Spratt & Spratt, 1964). In recent years the development of novel methods for the study of tumour proliferation by flow cytometry and immunohistochemistry have stimulated a renewed interest in the measurement of growth characteristics of human tumours. The most widely used method for the measurement of lung metastases in previous reports has been the graphical method on semi-logarithmic paper (Collins *et al.*, 1956; Schwartz, 1961). In this study, a more convenient and objective method based on linear regression is demonstrated, which may be done with an ordinary desk-top computer.

The reliability of measuremnent of tumour doubling times from chest x-rays has previously been only briefly discussed (Brenner *et al.*, 1967). In this study each metastasis was measured by two different investigators and the level of agreement assessed.

### Materials and methods

Between 1985 and 1990, 80 patients with lung metastases from soft-tissue or skeletal sarcomas were treated by the sarcoma group at the Department of Radiotherapy and Oncology, University of Helsinki. Twenty-three of these had bi-dimensionally measurable metastases in at least two sequential chest x-rays taken at least 14 days apart. The reason for exclusion was lack of follow-up in almost all excluded case. Patients on chemotherapy were included when at least one month had elapsed since the last chemotherapy cycle. No patients received chemotherapy during the period of measurement. The metastases were measured by two independent investigators (MT and TW). Only metastases considered measurable by both investigators were included in the calculations. Metastases, which according to the measurements did not grow in size (nine metastases in five patients with a median area of 1.3 $cm^2$) were excluded, which led to the exclusion of 2 patients from the study because none of the measured metastases seemed to increase in size during the follow-up period.

The final patient material consisted of 21 patients, and the total number of measured metastases was 52.

The largest longitudinal diameter ($d_1$) and the largest transverse diameter perpendicular to this ($d_2$) were measured from chest x-rays by the two investigators. In the measurements only PA (postero-anterior) views were utilised, since reliable measurements from lateral chest views were impossible to perform in a number of cases. In the calculations of cross-section area an elliptic shape of the metastases with an area of $(\pi{*}d_1{*}d_2)/4$ is postulated.

A regression analysis was performed on the logarithm of the product of the two perpendicular diameters versus time. A linear regression equation of the form $y = ax + b$ was calculated for each metastasis, where $y = \ln(d_1{*}d_2)$, $\ln$ = natural logarithm, $d_1$ = the longitudinal diameter, $d_2$ = the transverse diameter, a = the slope of the regression equation, x = the time from baseline, b = the constant term of the regression equation. The volume doubling time of each metastasis ($T_2$) was calculated from the slope (a) of the regression equation according to the formula $T_2 = 2\ln 2/_{3a}$.

The geometric mean of the estimates by the two investigators was used unless otherwise indicated. In patients with more than one metastasis the geometric mean of the doubling times of individual metastases was used as the patient-specific doubling time.

The statistical significance of the difference between inter- and intrapatient variation of tumour doubling times was studied with analysis of variance. The 95% range of agreement between the doubling time measurement by the two investigators was estimated from the standard deviation of the quotient of the two estimates under the assumption of a log-normal distribution (Brennan & Silman, 1992). The statistical significance of investigator bias in the estimates of the doubling time was tested with the one-sample *t*-test on the logarithm of the quotient of the two investigators' estimates.

The prognostic value of the doubling time for overall survival tested in a Cox analysis with the BMDP computer program (Dixon *et al.*, 1988).

### Results

The mean age of the patients was 47 years. Nine were females. Seven patients had previously received cytotoxic

Correspondence: C. Blomqvist, Department of Radiotherapy and Oncology, University of Helsinki, Haartmaninkatu 4, SF-00290 Helsinki, Finland.
Received 6 January 1993; and in revised form 9 April 1993.

**Table I** Clinical characteristics of the patients and estimated tumour doubling times

| *Sex and age* | *Histology* | *Grade* | *Site* | *Number of previous CT regimens* | *Number of metastases* | *Number of measurements* | *Total measurement time (months)* | $T_2$ *(days)* | $\Delta T_2$* (%) |
|---|---|---|---|---|---|---|---|---|---|
| M20 | Chondrosarcoma | 3 | Pelvis | 3 | 1 | 5 | 315 | 1172 | −65 |
| M21 | Osteosarcoma | 4 | Tibia | 0 | 1 | 3 | 35 | 50 | −15 |
| M27 | Sarcoma NOS | 4 | Neck | 1 | 6 | 5 | 77 | 39 | −41 |
| M29 | Sarcoma NOS | 4 | Axilla | 0 | 7 | 2 | 89 | 32 | −40 |
| M34 | Sarcoma Ewing | 4 | Maxilla | 1 | 1 | 4 | 27 | 28 | 15 |
| F35 | Leiomyosarcoma | 4 | Scapula | 3 | 1 | 2 | 26 | 11 | 20 |
| F36 | Sarcoma Ewing | 4 | Buttock | 0 | 1 | 3 | 39 | 16 | −6 |
| M37 | Sarcoma NOS | 4 | Buttock | 0 | 2 | 2 | 14 | 16 | −22 |
| F41 | Sarcoma NOS | 4 | Upper arm | 3 | 4 | 4 | 80 | 74 | −17 |
| M42 | MFH | 4 | Thigh | 0 | 3 | 3 | 20 | 9 | 0 |
| F51 | Schwannoma malignum | 3 | Retroperitoneum | 0 | 1 | 2 | 36 | 29 | 3 |
| F51 | Sarcoma NOS | 4 | Axilla | 0 | 1 | 2 | 232 | 54 | −2 |
| M54 | Liposarcoma | 4 | Upper leg | 0 | 2 | 2 | 84 | 35 | −11 |
| M55 | Liposarcoma | 3 | Shoulder | 0 | 1 | 3 | 91 | 75 | −7 |
| F56 | Leiomyosarcoma | 3 | Uterus | 1 | 5 | 4 | 55 | 37 | 15 |
| M61 | Sarcoma NOS | 4 | Maxilla | 0 | 1 | 2 | 34 | 37 | 5 |
| F63 | Sarcoma NOS | 4 | Foot | 0 | 1 | 3 | 43 | 26 | 21 |
| M64 | Sarcoma synoviale | 3 | Hip | 1 | 2 | 7 | 231 | 60 | 2 |
| M66 | Sarcoma NOS | 4 | Groin | 0 | 3 | 2 | 21 | 14 | −7 |
| F67 | MFH | 4 | Calf | 0 | 1 | 2 | 14 | 7 | −25 |
| F68 | Leiomyosarcoma | 2 | Retroperitoneum | 0 | 6 | 9 | 819 | 276 | −6 |

*Discrepancy in $T_2$ ($\Delta T_2$) estimated by two (MT and TW) independent investigators calculated as $[(T_2MT/T_2TW) - 1]*100$. M = male; F = female.

treatment. Patient characteristics, the number of measured metastases, the number of measurement time points, the time interval from the first to the last measurement and the calculated doubling times of the metastases in the individual patients is shown in Table I. The median metastasis doubling time for the 21 patients was 34.9 days and the geometric mean of $T_2$ was 36.9 days (range 7 to 1172 days). In a probit plot of the logarithm of $T_2$ the data points seemed to lie along a straight line ($r = 0.94$) indicating that the distribution was approximately log-normal. Under assumption of a log-normal distribution with a geometric mean of 36.9 days the estimated 95% range (tolerance interval) of doubling times was 3.9 to 352 days.

*Reproducibility of the measurements*

The doubling times measured by the two investigators showed were in good agreement in most cases. In a few cases, however, there was a considerable discrepancy between the estimates according to the measurements by the two observers (Table I). The estimates of the first investigators (MT) was in an average 16% lower than those of the second (TW). This difference was statistically significant ($P = 0.01$). The investigator obtaining the lower estimates of $T_2$ also obtained significantly smaller measurements of the sizes of the metastases (mean difference 7% or 0.41 $cm^2$, $P = 0.0001$).

**Figure 1** Growth measurements of metastases in twelve patients with more than two measurements of each metastasis. The patient identification code denotes sex (F = female, M = male) and age at primary diagnosis. $T_2$ are given in days. The scale of the abscissa (time) is linear and the scale of the ordinata (size) logarithmic.

**Figure 2** Correlation between the logarithm of $T_2$ and log-survival time from the detection of metastases.

The estimated 95% range for the quotient of the two $T_2$ measurements of the 52 individual metastases was 0.318 to 2.22. Thus the discrepancy of two independent estimates of $T_2$ are expected to lie between − 68% and + 122% in 95% of cases. The estimated 95% range for the patient specific $T_2$ calculated as the geometric mean of the doubling times of multiple metastases for each patient was narrower, − 53% to + 67%.

### *Variation in doubling times between different metastases in the same patients compared to variation between patients*

Eleven patients had more than one measurable metastasis. The variance in $T_2$ significantly ($P = 0.0001$) larger between the patients (mean square of natural logarithm of the doubling time between patients 3.6) than within each patient (mean square of ln doubling time within patients 0.43)

### *Evidence of exponential growth in patients with three or more measurements*

Twenty-seven metastases in 12 patients were measurable more than twice (Figure 1). Linear regression of the logarithms of the sizes of these metastases yielded linear correlations very close to unity in most cases ($r = 0.66$ to 0.998, median 0.987).

### *Correlation between doubling time and survival*

There was a positive correlation ($r = 0.55$) between metastasis doubling time and the survival time from the diagnosis of metastatic disease (Figure 2). In a Cox proportional hazard model, however, the tumour doubling time did not attain statistical significance as a prognostic variable for survival time ($P = 0.10$, no other factors included in the model).

### Discussion

Charbit *et al.*, reviewed the literature on the growth rate of human tumours, and found a total of 87 published cases of mesenchymal tumours (Charbit, *et al.*, 1971). Since that only a few additional series have been published on measurement of $T_2$ in metastases from sarcomas (Band & Kocandrle, 1975; Chaninian, 1972; Rööser, *et al.*, 1987). Most previous series are small, the only study larger than the present one is the series of Breur et al. (Breur, 1966).

The sarcoma group at the University of Helsinki has been treating bone sarcoma patients on a centralised fashion since 1981 and soft-tissue sarcomas since 1987. Approximately five new cases of bone sarcomas and 50 new soft-tissue sarcomas are seen yearly. After primary treatment the patients are followed up with two to six months intervals and chest x-rays are taken at each visit. This has provided a representative patient material to study the growth characteristics of lung metastases. Despite the relatively intense follow-up only about one fourth of the patients with lung-metastases were found to have measurable lesions not undergoing treatment and visible in at least two chest x-rays.

In previous studies, the graphic method has been the most commonly used (Brenner *et al.*, 1967; Collins *et al.*, 1956; Rööser *et al.*, 1987). This method has the drawback of being prone to bias, since the straight lines adjoining the measurement points are estimated by eye-sight. In our study a more objective method based on linear regression analysis is used. This method can be performed with commonly available statistical computer programs. We are aware of only one previous publication of this method in which, however, the methodology is only briefly outlined (Mattson *et al.*, 1980). In the present study the size of the metastases were measured only on PA chest views, since the measurement of the sagittal diameter proved difficult in many cases. It can be shown by elementary calculation that volume doubling time can be calculated from cross-section areas without estimation of the third diameter providing the shape of the tumour remains constant.

In the review by Charbit *et al.* a median $T_2$ of malignant mesenchymal tumours of 37 days and a geometric mean of 41.4 days was observed in 87 patients (Charbit *et al.*, 1971). These figures agree very well with ours. As in previous studies the distribution of $T_2$ was approximately log-normal (Charbit *et al.*, 1971; Spratt, 1965; Spratt & Spratt, 1964). The span of interpatient variation of $T_2$ in this patient material was almost 100-fold, and an estimated 95% range of the same magnitude. The wide range of doubling times in sarcomas is probably one of the factors responsible for the large variation in the clinical behaviour of these tumours. Like previous investigators (Brenner *et al.*, 1967; Rööser *et al.*, 1987) we found that the variation in $T_2$ between individual patients was considerably larger than between different metastases in the same patient. Statistical testing of this difference showed it to be highly significant, providing formal evidence for this previously reported finding.

There was a positive correlation between the logarithm of $T_2$ and the logarithm of survival time from detection of metastases. The scatter around the regression line was, however, considerable, and the coefficient of determination ($r^2$) was only 0.30. Thus differences in $T_2$ explained only one third of the variation of the survival times from detection of metastatic disease. $T_2$ has previously been found to correlate well with the prognosis in both sarcomas (Joseph *et al.*, 1971) and lung tumours of other histology (Mattson & Holsti, 1980) and in sarcoma patients after resection of lung metastases (Spratt & Spratt, 1964). When tested as a prognostic factor for survival with a conventional Cox model $T_2$ did, however, not reach statistical significance in this study, possibly because of the small sample size. Even if metastasis doubling time may be a valuable prognostic factor for outcome in metastatic lung disease its practical use is diminished by the fact that only a minority of patients (in this study one fourth) have metastases that are reliably measurable.

In animal studies it has been shown that many tumours do not grow in a constant exponential fashion, and that $T_2$ is retarded when the tumour grows in size; this is referred to as Gompertzian growth (Laird, 1964; Laird, 1965). In human tumours, however, no visible evidence of Gompertzian growth of lung metastases has been found by previous investigators (Brenner *et al.*, 1967; Breur, 1966; Rööser *et al.*, 1987; Schwartz, 1961). Twelve patients in this study had measurements more than twice. No evidence of Gompertzian growth was found in these patients; the growth of most metastases seemed to be well described by an exponential function during the whole observation period, seen as a high linear correlation of the logarithm of metastatic area with time. The reason for the failure to detect Gompertzian growth in human tumours is unclear, but may partly be a reflection of the limited potential follow-up times in human tumours.

Little data are available on the reproducibility of measurements of growth rate of neoplastic lung lesions. Brenner has estimated the error of measurements of $T_2$ of lung metastases to be 11% or less, assuming a follow-up time twice the tumour doubling time and measurements from six different radiographs (Brenner *et al.*, 1967). In a human patient material the need for early treatment precludes such a long follow-up in most cases. In this study measurements were performed by two independent investigators. The disagreement between the two estimates of $T_2$ of individual metastases was surprisingly large. The repeatability of the patient specific $T_2$ was better. Most of the disagreement originated from a few patients with small poorly visible metastases. Moreover, the four- to seven-fold range of disagreement in estimates is still small compared to the 100-fold variation of $T_2$ between different patients. Interestingly one of the two investigators seemed to obtain significantly lower values of $T_2$ than the other, even if the systematic bias was negligibly small. This, however, underscores the necessity of having all measurements done by the same investigator in repeated measurements.

The authors wish to thank professor Mårten Brenner for valuable comments on the mathematical methodology.

### References

BAND, P.R. & KOCANDRLE, C. (1975). Growth rate of pulmonary metastases in human sarcomas. *Cancer*, **36**, 471–474.

BRENNAN, P. & SILMAN, A. (1992). Statistical methods for assessing observer variability in clinical measures. *B M J*, **304**, 1491–1494.

BRENNER, M.W., HOLSTI, L.R. & PERTTALA, Y. (1967). The study by graphical analysis of the growth of human tumors and metastases of the lung. *Br. J. Cancer*, **21**, 1–13.

BREUR, K. (1966). Growth rate and radiosensitivity of human tumours. *Europ. J. Cancer*, **2**, 158.

CHANINIAN, P. (1972). Relationship between tumor doubling time and anatomoclinical features in 50 measurable pulmonary cancers. *Chest*, **61**, 340–345.

CHARBIT, A., MALAISE, E.P. & TUBIANA, M. (1971). Relation between the pathological nature and the growth rate of human tumors. *Europ. J. Cancer*, **7**, 307–315.

COLLINS, V.P., LOEFFLER, R.K. & TIVEY, H. (1956). Observations on growth rates of human tumors. *Cancer*, **76**, 988–1000.

DIXON, M.B., ENGELMAN, L., HILL, M.A. & JENNRICH, R.I. (1988). *BMDP Statistical Software Manual*. University of California Press: Berkeley Los Angeles London.

JOSEPH, W.L., MORTON, D.L. & ADKINS, P.C. (1971). Prognostic significance of tumor doubling time in evaluating operability in pulmonary metastatic disease. *J. Thoracic Cardiovascular Surg.*, **61**, 23–32.

LAIRD, A.K. (1964). Dynamics of tumor growth. *Br. J. Cancer*, **18**, 490–502.

LAIRD, A.K. (1965). Dynamics of tumour growth: Comparison of growth rates and extrapolation of growth curve to one cell. *Br. J. Cancer*, **19**, 278–291.

MATTSON, K. & HOLSTI, L.R. (1980). Prognostic value of doubling time in lung cancer. *Strahlenterapie*, **156**, 632–636.

MATTSON, K., SALMO, M. & HOLSTI, L.R.H. (1980). Accuracy of determination of doubling time in lung cancer. *Br. J. Cancer*, **41, Suppl IV**, 49.

RÖÖSER, B., PETTERSSON, H. & ALVEGÅRD, T. (1987). Growth rate of pulmonary metastases from soft tissue sarcoma. *Acta Oncol.*, **26**, 189–192.

SCHWARTZ, M. (1961). A biomathematical approach to clinical tumor growth. *Cancer*, **14**, 1272–1294.

SPRATT, J.S. (1965). The rates of growth of skeletal sarcomas. *Cancer*, **18**, 14–24.

SPRATT, J.S. & SPRATT, T.L. (1964). Rates of growth of pulmonary metastases and host survival. *Ann. Surgery*, **159**, 161–171.